# Diagnostic and Prognostic Value of Neutrophil Extracellular Trap Levels in Patients With Acute Aortic Dissection

**DOI:** 10.3389/fcvm.2021.683445

**Published:** 2022-02-15

**Authors:** Shuofei Yang, Yongsheng Xiao, Yuanfeng Du, Jiaquan Chen, Qihong Ni, Xiangjiang Guo, Guanhua Xue, Xupin Xie

**Affiliations:** ^1^Department of Vascular Surgery, Renji Hospital, School of Medicine, Shanghai Jiaotong University, Shanghai, China; ^2^Department of Vascular Surgery, Tianjin 4th Centre Hospital, The Fourth Central Hospital Affiliated to Nankai University, The Fourth Center Clinical College of Tianjin Medical University, Tianjin, China; ^3^Department of Neurosurgery, School of Medicine, Affiliated Hangzhou First People's Hospital, Zhejiang University, Hangzhou, China; ^4^Department of Vascular Surgery, School of Medicine, Affiliated Hangzhou First People's Hospital, Zhejiang University, Hangzhou, China

**Keywords:** neutrophil extracellular trap, acute aortic dissection, serum biomarker, diagnostic marker, prognostic marker

## Abstract

**Background:**

Acute aortic dissection (AAD) is a fatal disease demanding prompt diagnosis and proper treatment. There is a lack of serum markers that can effectively assist diagnosis and predict prognosis of AAD patients.

**Methods:**

Ninety-six AAD patients were enrolled in this study, and 249 patients with chest pain due to acute myocardial infarction, pulmonary embolism, intramural hematoma, angina or other causes and 80 healthy controls were included as control group and healthy control group. Demographics, biochemical and hematological data and risk factors were recorded as baseline characteristics. The 1-year follow-up data were collected and analyzed. The diagnostic performance and ability to predict disease severity and prognosis of NET components in serum and aortic tissue were evaluated.

**Results:**

Circulating NET markers, citH3 (citrullination of histone 3), cell-free DNA (cfDNA) and nucleosomes, had good diagnostic value for AAD, with superior diagnostic performance to D-dimer in discriminating patients with chest pain due to other reasons in the emergency department. Circulating NET marker levels (i.e., citH3, cfDNA and nucleosomes) of AAD patients were significantly higher than that of control group and healthy control group. In addition, circulating NET markers levels were closely associated with the disease severity, in-hospital death and 1-year survival of AAD patients. Systolic blood pressure < 90 mmHg and serum citH3 levels were identified as independent risk factors for 1-year survival of AAD patients. Excessive NET components (i.e., neutrophil elastase and citH3) in the aortic tissue of AAD patient were significantly higher than that of healthy donor aortic tissue. The expression levels of granules and nuclear NET components were significantly higher in aortic tissue from AAD patients than controls.

**Conclusions:**

Circulating NET markers, citH3, cfDNA and nucleosomes, have significant diagnostic value and predictive value of disease severity and prognosis of AAD patients. The NETs components may constitute a useful diagnostic and prognostic marker in AAD patients.

## Introduction

Acute aortic dissection (AAD) is a fatal aortic disease with high mortality and morbidity that demands prompt diagnosis and proper treatment (1). Despite recent advances in diagnostic imaging methods, AAD remains a challenge to diagnose. A widely available and cost-effective measure such as a blood test that can rule in and/or rule out the disease would indeed aid in quick diagnosis, benefiting patients and caregivers. Several major diseases that cause chest pain, such as acute myocardial infarction (AMI), pulmonary embolism (PE), intramural hematoma and angina, require differential diagnosis with AAD. Moreover, there is still a lack of effective markers to accurately predict the in-hospital and long-term outcomes of patients with AAD after surgical repair.

Neutrophils are the most abundant cell type in leukocytes and play a crucial role in the innate immune system (2). Neutrophils are also involved in the pathological mechanism of aortic dissection (AD) (3). AAD is initiated by neutrophil infiltration of the aortic intima, and local neutrophil recruitment and activation in response to AAD can lead to aortic rupture (4, 5). In patients with AAD receiving surgical repair, the neutrophil to lymphocyte ratio may be used to predict worse outcomes and hospital mortality (6). In the inflammatory response, neutrophils play critical roles through the release of neutrophil extracellular traps (NETs), which are extracellular neutrophil-derived web-like structures that constitute a DNA backbone containing histones and neutrophil granule proteins. A critical step in NET release is the citrullination of histone 3 (citH3), a process mediated by protein deiminase 4, and citH3 is considered one of the most specific markers for NET formation assessment (7). Initially, NETs were thought to provide defense against pathogens (8). In recent years, NETs have been implicated in a number of cardiovascular diseases (9). By immunophenotypic analysis, NETs were found to participate in the tissue repair of AD (10).

NETs could be used as a new circulating marker for several cardiovascular diseases, such as acute coronary syndrome, acute ischemic stroke, myocardial infarction, and deep venous thrombosis (11–13). Data on NET presence in the serum and tissue of AAD patients and the association between NET levels and clinical outcomes are scarce. Hence, this study sought to determine whether NETs may serve as disease biomarkers in AAD patients. Specifically, our aims were to examine the diagnostic value of NETs for AAD and the predictive significance of NETs for disease severity, in-hospital mortality and 1-year survival in AAD patients receiving surgical repair.

## Patients and Methods

### Patients

The study was approved by the ethical committee of Renji Hospital, School of Medicine, Shanghai Jiaotong University (No. RA2020-253). All patients or their proxies provided written informed consent. Ninety-six consecutive patients with AAD hospitalized in Renji Hospital between May 01, 2016, and April 04, 2019, were enrolled in this study. Diagnoses were made based on computed tomography angiography (CTA), digital subtraction angiography and, when appropriate, magnetic resonance angiography (MRA). The Stanford classifications of AAD were evaluated at the time of diagnosis. Stanford type A aortic dissection (TAAD) involves the ascending aorta, whereas type B aortic dissection (TABD) involves the descending aorta only. The severity of disease was measured by the Acute Physiology, Age, Chronic Health Evaluation II (APACHE II) score at hospital entrance and discharge. The detection risk score of AD was calculated at admission to the emergency department according to the guidelines (14). During the same time period, 249 patients admitted to the emergency chest pain center with a diagnosis of AMI, PE, angina or others were included in the control group. In addition, 80 healthy controls were included in this study. Demographics, biochemical and hematological data, clinical history, and risk factors were recorded as baseline patient characteristics. Clinical follow-up was performed 1 year after hospital discharge in all AAD patients. This was conducted by reviewing the electronic records in clinics or by telephone contact. Follow-up records included reoperation information, CTA or MRA follow-up information, mortality, and cause and date of death.

Citrated-anticoagulated venous blood was obtained from all the patients within 3 h of diagnosis and on the last morning before discharge. Platelet-poor plasma was prepared by centrifugation of the blood (2,500 × g) for 10 min at 22°C, and the plasma was stored at −80°C until analysis. Aortic tissue samples were obtained from 45 AAD patients included in this study who received open surgery. Twenty normal aortic tissues were obtained from organ donors (crash victims or brain-dead patients). Tissue samples were stored at −80°C until analysis.

### NET Markers

We evaluated three different markers of NETs [i.e., cfDNA (cell-free DNA), nucleosomes and citH3]. citH3 is currently considered to be the most specific marker, as H3 citrullination is required for chromatin decondensation in neutrophils. Detection of cfDNA and nucleosomes was performed as described previously (13). For cfDNA determination, plasma was diluted 1:10 with phosphate-buffered saline and mixed with an equal volume of 1 mM SytoxGreen (Invitrogen, Carlsbad, CA, USA; No. S7020) in PBS. Fluorescence was determined in a fluorescence microplate reader (Gemini XPS; Molecular Devices, Sunnyvale, CA, USA). A calibration curve was generated with calf thymus DNA (Invitrogen; No. 15633019) in PBS. Nucleosomes were measured with the Cell Death Detection ELISAPLUS kit (Roche Diagnostics, Madrid, Spain; No. 11774425001) according to the manufacturer's instructions. Determination of citH3 was performed as previously described (15). Briefly, plasma samples were mixed with a monoclonal mouse anti-histone biotinylated antibody in a streptavidin-coated plate. A rabbit polyclonal anti-histone-H3 (citrullinated R17 + R2 + R8) (Abcam Inc., MA, USA; No. ab81797) antibody was used in the second step. Detection was performed with a peroxidase-linked antibody (GE Biosciences, Barcelona, Spain; No. A1783). Values were normalized to a pool of samples from normal subjects. Values are expressed as individual absorption values. The neutrophil elastase (NE) concentration was measured using commercially available ELISA kits.

### Proteomics Analysis

In this study, we built a custom pathway of NET-associated proteins as described previously (16). To build a custom “NETosis” pathway, twenty-three proteins that belonged to five subcellular compartments (nucleus, granules, cytoplasm/cytoskeleton, enzymes, and plasma membrane) were identified by a literature screen for detailed characterization of NET proteins (17, 18). According to a Gene Set Enrichment Analysis heat map, the custom NETosis pathway was enriched in aortic tissue from AAD patients vs. normal controls. Samples were reduced, alkylated and trypsin-digested according to the iTRAQ manufacturer's instructions (AB Sciex Inc., MA, USA). To diminish any potential variation introduced by the labeling reaction, samples from AAD patients and normal controls were split into two aliquots of 60 μg to perform two technical replicates with tag swapping. Each peptide solution was labeled at room temperature for 1 h with one iTRAQ reagent vial. To verify the labeling efficiency, 1 μg of each labeled sample was individually analyzed by liquid chromatography-tandem mass spectrometry (LC-MS/MS) as specified below. Acquired data were searched against the Mascot database, setting iTRAQ labeling as the variable modification. No unmodified peptides were identified from the search, and all the peptides were correctly modified at the N-terminus and at each lysine residue. Finally, the four iTRAQ-labeled samples were combined in a 1:1:1:1 ratio, and the pool was vacuum dried in a SpeedVac system.

### Immunofluorescence

NET identification in tissue samples was performed by immunofluorescence staining. The NE/citH3 pair was researched in paraffin-embedded, 3-μm-thick sections. The slides were incubated with the primary antibodies (anti-NE antibody, MAB91671, R&D Systems, Minneapolis, USA; anti-H3Cit antibody, ab5103, Abcam, Cambridge, UK; both 1:50 dilution) at 4°C overnight after blocking with goat serum. Then, the sections were incubated with secondary antibodies (Alexa Fluor 488, green, ab150077; Alexa Fluor 647, red, ab150075; both from Abcam, Cambridge, UK) for 1 h at room temperature. DAPI was used for nuclear staining (ZSGB Biotech, Beijing, China; No. ZLI-9577). The slides were analyzed with a confocal laser scanning microscope (TCS-SP5; Leica, Wetzlar, Germany). The average numbers of NE and H3Cit double-positive cells were calculated by two independent researchers counting five random microscopic fields.

### Statistics

Data are expressed as the means ± SEM of absolute values or as percentages. Continuous variables were analyzed with the Mann-Whitney test. Discrete variables were evaluated with a contingency χ^2^ test. By the Shapiro-Wilk test, the value of citH3, cfDNA and nucleosomes were found to be normally distributed. The Spearman coefficient (*r*) was used to quantify the correlations between variables. Compared with D-dimer, the diagnostic performance of NET markers for distinguishing AAD from all other diseases, AMI, PE, or angina was assessed using receiver operating characteristic curve (ROC) analysis. The area under the ROC (AUR), sensitivity, specificity, accuracy, positive predictive value, and negative predictive value were calculated. The Wald test was used to assess the significance of the difference between areas under the ROC curve. The optimal cutoff point from the study was the threshold leading to the maximum summation of sensitivity and specificity. Univariate logistic regression analysis was used to assess the association between risk factors and in-hospital death or 1-year survival, and a multivariate Cox regression analysis was performed using variables with *P* ≤ 0.20 in univariate analysis. A two-sided *P*-value <0.05 was considered statistically significant. Statistical analyses were performed with SPSS 20 (SPSS, Inc., Chicago, IL, USA).

## Results

### Baseline Demographic Information and Hematological Parameters

A total of 96 patients, 42 TAADs and 54 TBADs, were enrolled in the AAD group. Another 249 patients were enrolled in the control group, including 10 IH cases, 63 AMI cases, 42 PE cases, 66 angina cases and 68 cases with other causes. The baseline demographic information, medical history, risk factors, and biochemical and hematological data are shown in Table 1. The demographic information, biochemical and hematological data of 80 healthy controls are shown in Supplementary Table 1. The rates of hypertension and Marfan syndrome were significantly higher in the AAD group than in the control group. Moreover, AAD patients had significantly higher levels of D-dimer than patients in the control group. There was no significant difference between the two groups regarding other parameters. The AAD patients were stratified according to classical risk factors. No significant differences among any of the three NET markers were observed between patients with or without hypertension, diabetes, smoking history and atrial fibrillation. The cfDNA levels in patients with Marfan syndrome were significantly higher than those in patients without Marfan syndrome. However, the levels of citH3 and nucleosomes were comparable between patients with or without Marfan syndrome (Table 2).

**Table 1:** Baseline characteristics of chest pain patients included in this study.

**Characteristics**	**AAD** **(*n* = 96)**	**Controls(*n* = 249)**	***P*-value**
Age, mean (range)	59.1 (35–85)	60.3 (40–92)	0.45
Male sex, *n* (%)	53 (55.2)	128 (51.4)	0.53
***Medical history and risk factors, n (%)***				
Hypertension	72 (75.0)	129 (51.8)	<0.05
Diabetes	44 (45.8)	109 (43.8)	0.62
Stroke	28 (29.2)	80 (32.1)	0.60
Hyperlipidemia	48 (50.0)	135 (54.2)	0.48
Smoking	55 (57.3)	125 (50.2)	0.24
Marfan syndrome	12 (12.5)	1 (0.4)	<0.01
Atrial fibrillation	28 (29.2)	63 (25.3)	0.47
Valvulopathy	20 (20.8)	42 (16.9)	0.39
***Diagnosis, n (%)***				
TAAD	42 (43.8)	/	/
TBAD	54 (56.3)	/	/
IH	/	10 (4.0)	/
AMI	/	63 (25.3)	/
PE	/	42 (16.9)	/
Angina	/	66 (26.5)	/
Others	/	68 (27.3)	/
***Biochemical and hematological data, mean ±SD***				
Glucose, mg/dL	130.3 ± 52.4	125.4 ± 44.2	0.42
Creatinine, μmol/L	148.7 ± 33.6	142.9 ± 40.7	0.36
Uric acid, mg/dL	6.1 ± 2.4	5.9 ± 2.9	0.37
Cholesterol, mg/dL	178.3 ± 39.2	183.2 ± 29.9	0.72
Triglycerides, mg/dL	182.1 ± 88.4	189.1 ± 79.2	0.60
AST, U/L	28.2 ± 13.4	30.2 ± 20.2	0.13
ALT, U/L	38.3 ± 16.7	39.6 ± 26.3	0.22
Bilirubin, mg/dL	1.9 ± 1.6	1.8 ± 0.8	0.82
Hematocrit, %	41.3 ± 6.3	39.4 ± 5.6	0.63
Platelets, 10^3^/μL	248.3 ± 56.4	256.6 ± 47.2	0.20
Leukocytes, 10^3^/μL	6.4 ± 3.4	6.8 ± 3.9	0.74
Lymphocytes, %	26.3 ± 9.2	27.5 ± 8.4	0.20
Neutrophils, %	74.4 ± 18.3	73.9 ± 19.8	0.48
Monocytes, %	8.8 ± 4.2	9.0 ± 7.3	0.33
D-dimer, ng/ml	2080.5 ± 1131.9	1867.3 ± 2007.3	<0.05

*AAD, acute aortic dissection; TAAD, type A aortic dissection; TBAD, type B aortic dissection; IH, intramural hematoma; AMI, acute myocardial infarction; PE, pulmonary embolism; SD, standard deviation; AST, aspartate transaminase; ALT, alanine aminotransferase*.

**Table 2:** NETs and risk factors before the onset of the acute event.

**Characteristics**		**citH3 (ng/ml)**	***P***	**cfDNA (AU)**	***P***	**Nucleosomes (AU)**	***P***
Hypertension	Yes	0.46 ± 0.23	0.73	698.82 ± 306.28	0.11	1.56 ± 0.82	0.82
	No	0.47 ± 0.26		788.13 ± 438.75		1.58 ± 0.88	
Diabetes	Yes	0.46 ± 0.23	0.56	692.48 ± 317.45	0.31	1.53 ± 0.85	0.75
	No	0.47 ± 0.25		745.40 ± 365.98		1.59 ± 0.83	
Smoking	Yes	0.48 ± 0.24	0.12	749.98 ± 355.40	0.21	1.61 ± 0.85	0.32
	No	0.44 ± 0.24		682.46 ± 328.01		1.50 ± 0.82	
Marfan syndrome	Yes	0.47 ± 0.21	0.52	664.25 ± 210.41	0.04	1.52 ± 0.74	0.21
	No	0.47 ± 0.24		729.27 ± 359.05		1.57 ± 0.85	
Atrial fibrillation	Yes	0.49 ± 0.25	0.44	757.68 ± 359.99	0.09	1.57 ± 0.80	0.71
	No	0.45 ± 0.24		706.10 ± 338.55		1.56 ± 0.85	

*citH3, citrullination of histone 3; cfDNA, cell-free DNA; AU, arbitrary units*.

### Diagnostic Performance of Circulating NET Markers for Discriminating AAD

**Figure 1:**
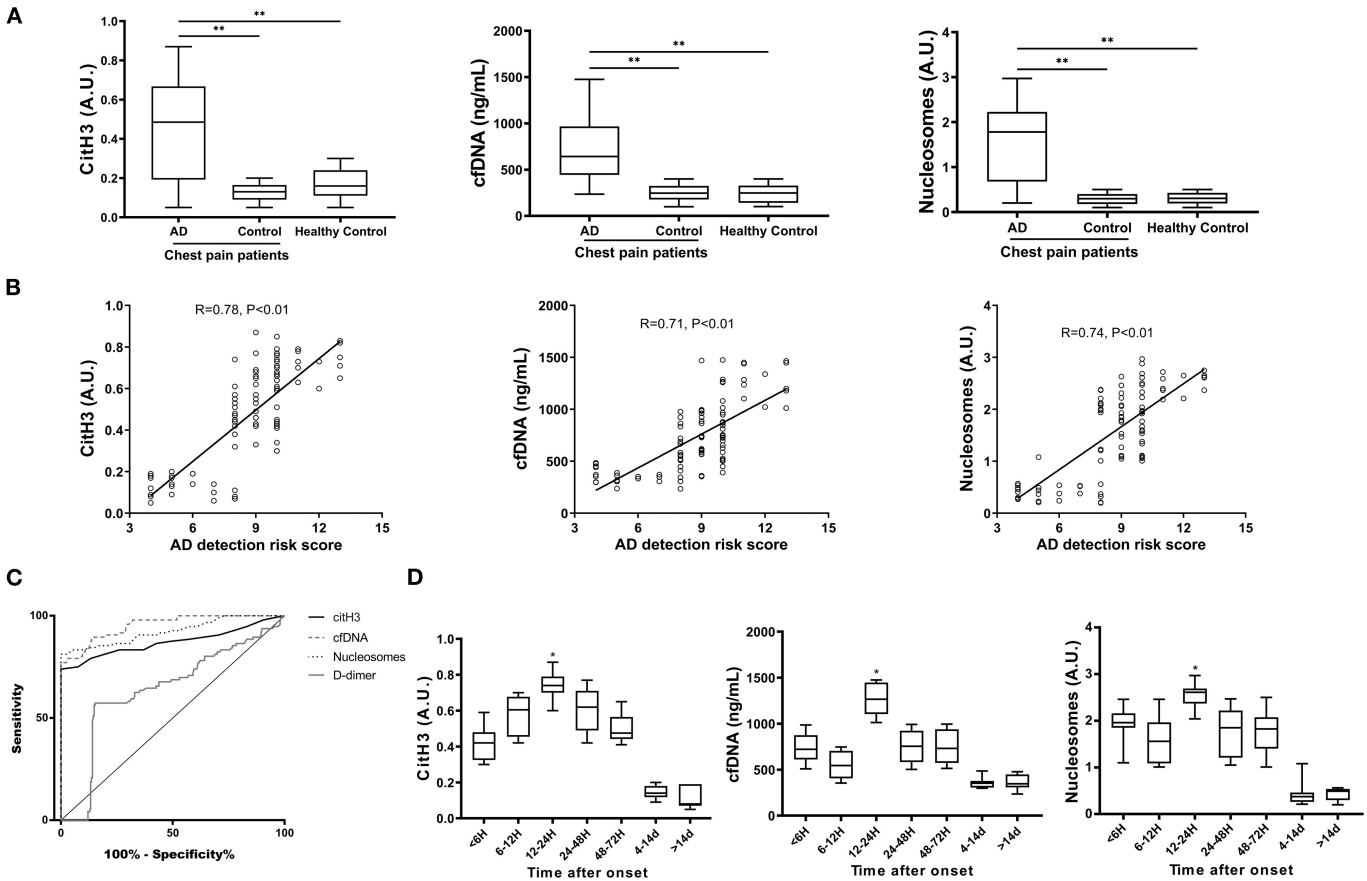
The diagnostic performance of circulating NET markers for discriminating AAD. **(A)** Serum levels of citrullinated H3 (citH3), cell-free DNA (cfDNA), and nucleosomes were evaluated in 96 patients with AAD and 249 patients in the control group. CitH3 and nucleosomes are expressed in arbitrary units (AU). The cfDNA concentration (ng/mL) was determined based on a calibration curve of calf thymus DNA. All three markers were significantly higher in the AAD group than in the control group. **(B)** Serum levels of citH3, cfDNA and nucleosomes were positively correlated with the detection risk score of AD. **(C)** ROC curve for the diagnosis of AAD. Circulating NET markers showed superior overall diagnostic performance compared with D-dimer when sudden-onset chest pain was present in the emergency department. **(D)** The time course of NET markers was examined in patients with AAD according to the admission time from symptom onset. The peak NET marker levels occurred within 12–24 h of symptom onset. ^*^P < 0.05, ^**^P < 0.01.

The values of citH3, cfDNA and nucleosomes in patients with AAD were significantly higher than those in the control group or healthy controls (Figure 1A) Circulating levels of citH3, cfDNA, and nucleosomes were positively correlated with the detection risk score of AD (Figure 1B). In the ROC curve, the AURs in patients with AAD vs. all control patients were 0.87 for citH3, 0.95 for cfDNA, 0.92 for nucleosomes, and 0.64 for D-dimer (Figure 1C). Thus, circulating NET markers showed superior overall diagnostic performance compared with D-dimer when sudden-onset chest pain was present in the emergency department. cfDNA at cutoff levels of 403.5 ng/ml and D-dimer at 2015 ng/ml were the thresholds leading to the maximum summation of sensitivity and specificity in discriminating AAD from all other diagnoses. The corresponding sensitivities were 77.08% for cfDNA and 57.29% for D-dimer, and the specificities were 100% for cfDNA and 84.74% for D-dimer, resulting in 93.62% of patients for cfDNA and 55.61% of patients for D-dimer being correctly classified (Supplementary Table 2).

The time course was also examined in the AAD group using box plot analysis according to the admission time from symptom onset. The peak NET marker levels occurred within 12–24 h after symptom onset (Figure 1D). In addition, no significant difference in circulating NET markers was found between patients with TAAD and TBAD or among different subsets of the control group. There was no correlation between age and circulating levels of NET markers (Supplementary Figure 1).

### Association Between Circulating NET Markers and Disease Severity of AAD at Onset and Discharge

**Figure 2:**
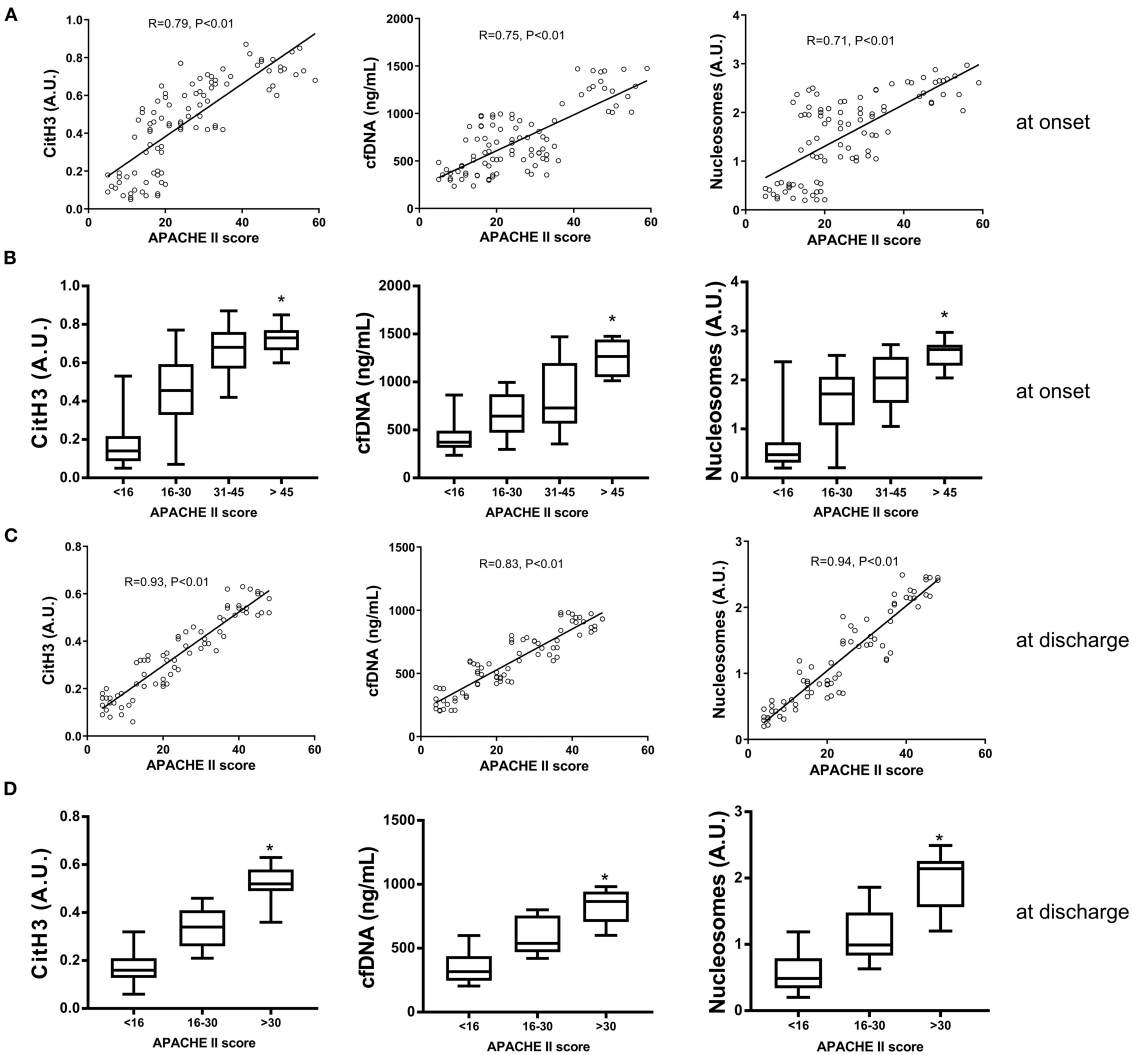
The association between NET markers and disease severity of AAD at onset and discharge. **(A)** Serum levels of citH3, cfDNA and nucleosomes were positively correlated with the APACHE II score at disease onset. **(B)** AAD patients were classified into four groups according to APACHE II score. Patients with higher scores had significantly higher levels of all three NET markers. **(C)** The levels of all three NET markers were positively correlated with the APACHE II score at discharge. **(D)** AAD patients with greater disease severity showed significantly higher levels of NET markers. ^*^P < 0.05.

There was a positive correlation between the APACHE II score and the levels of all three NET markers at disease onset (Figure 2A). When patients were classified according to the APACHE II score into four groups, those with higher scores had significantly higher levels of all three NET markers (Figure 2B). At discharge, the levels of all three NET markers were also positively correlated with the APACHE II score (Figure 2C). Classified by APACHE II score into three groups, patients with greater disease severity showed significantly higher levels of the three NET markers (Figure 2D). These results demonstrated the association between circulating levels of NET markers and disease severity at both admission and discharge.

### Prognostic Significance of Circulating NET Markers for Predicting In-hospital Death and 1-year Survival of Patients With AAD

**Figure 3:**
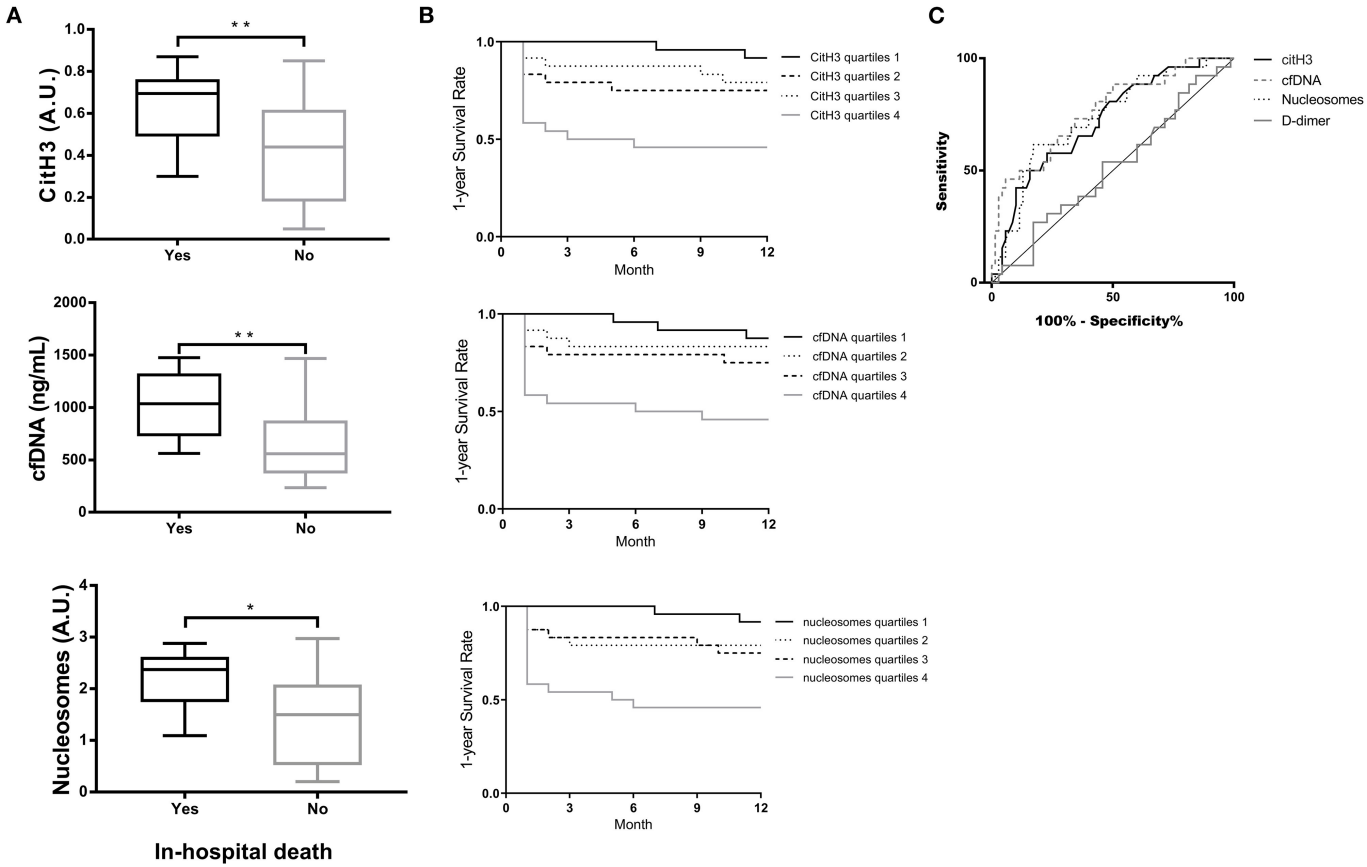
Prognostic significance of circulating NET markers for predicting in-hospital death and 1-year survival of AAD patients. **(A)** Serum levels of citH3, cfDNA and nucleosomes in patients with in-hospital death were significantly higher than those in patients without in-hospital death. **(B)** AAD patients with the highest quartiles of citH3, cfDNA or nucleosome levels presented significantly lower survival rates by 1 year than patients with the lower three quartiles of citH3. **(C)** Based on the ROC curve, three circulating NET markers showed superior prediction ability of 1-year survival compared with D-dimer. ^*^P < 0.05, ^**^P < 0.01.

The results in Figure 3A show that the levels of NET markers in patients with in-hospital death were significantly higher than those in patients without in-hospital death. AAD patients with the highest quartiles of citH3, cfDNA, or nucleosome levels presented significantly lower survival rates by 1 year than patients with the lower three quartiles of citH3 (Figure 3B). Based on the ROC curve, three circulating NET markers showed superior predictive ability for 1-year survival compared with D-dimer (Figure 3C). The AURs were 0.72 for citH3, 0.76 for cfDNA, 0.73 for nucleosomes, and 0.51 for D-dimer. The cfDNA at cutoff levels of 1,052 ng/ml and D-dimer at 915 ng/ml were threshold values. The corresponding sensitivities were 46.15% for cfDNA and 26.92% for D-dimer, and the specificities were 94.29% for cfDNA and 82.86% for D-dimer (Supplementary Table 3).

### Risk Factor Analysis of 1-year Survival in AAD Patients

**Figure 4:**
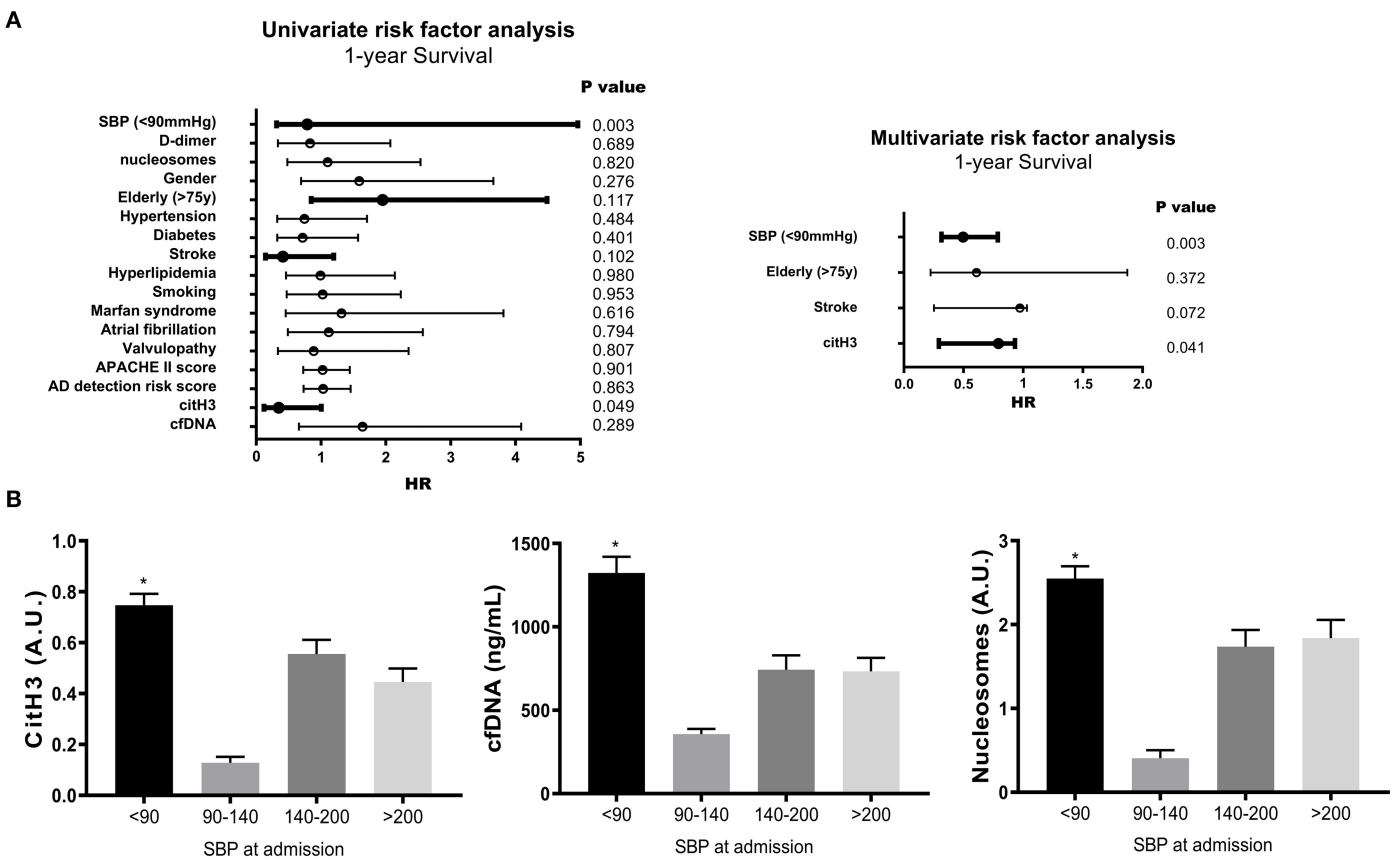
Risk factor analysis of 1-year survival in AAD patients. **(A)** By univariate and multivariate risk factor analysis, systolic blood pressure (SBP) < 90 mmHg and citH3 levels were identified as independent risk factors for 1-year survival. **(B)** AAD patients with SBP < 90 mmHg had significantly increased circulating NET markers compared with patients with SBP > 90 mmHg. ^*^P < 0.05.

We next examined whether NET markers were independently associated with the 1-year survival of AAD patients. By univariate and multivariate risk factor analysis, systolic blood pressure (SBP) < 90 mmHg and citH3 levels were identified as independent risk factors for 1-year survival (*p* < 0.05) (Figure 4A and Supplementary Table 4). AAD patients with SBP < 90 mmHg had significantly increased circulating NET markers compared with patients with SBP > 90 mmHg (Figure 4B).

### Excess NET Components in Aortic Tissue and Their Association With Disease Severity and Prognosis in Patients With AAD

**Figure 5:**
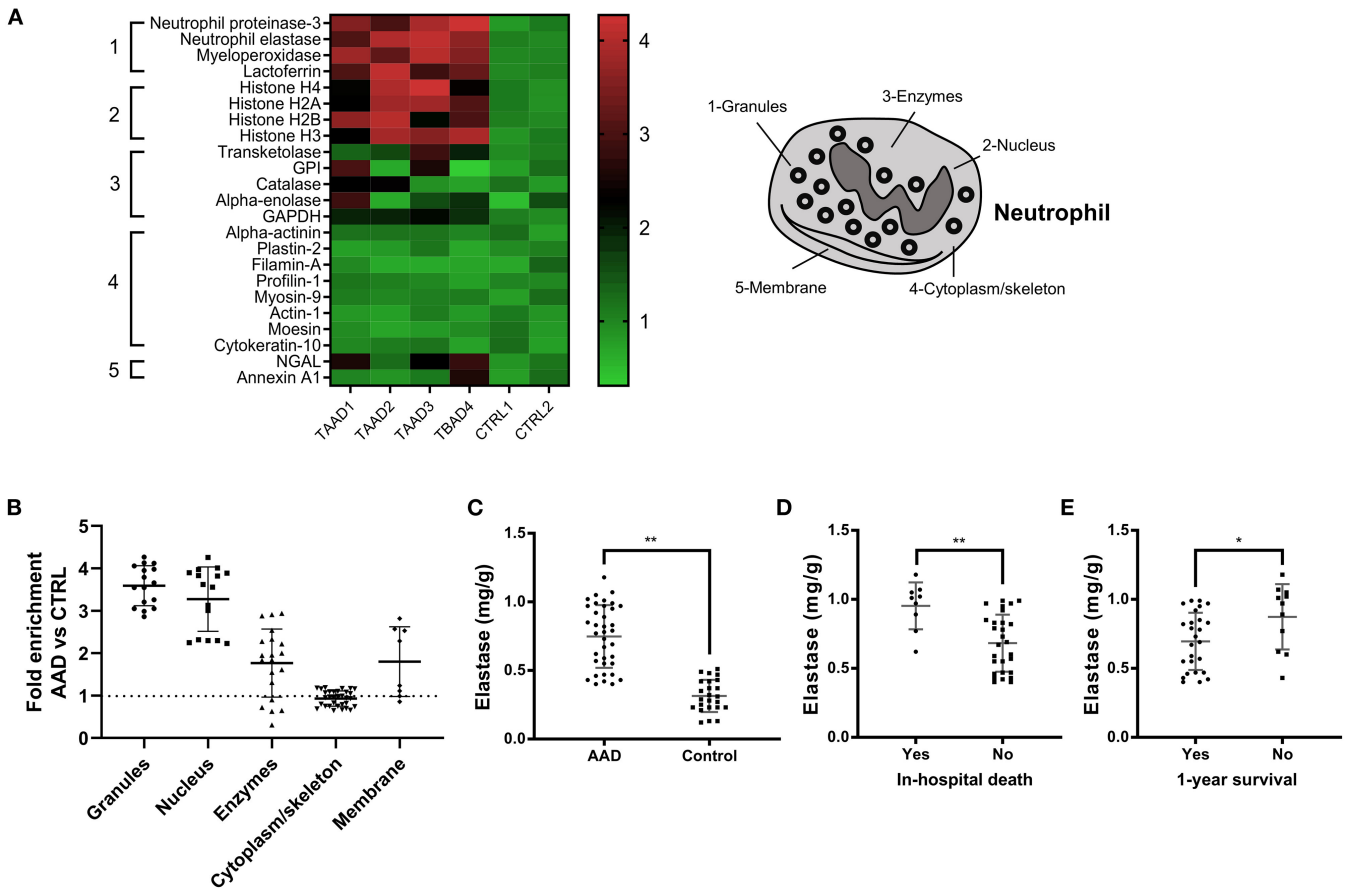
Custom proteomic analysis of aortic tissue lysates from AAD patients. **(A)** Twenty-three proteins that belonged to five subcellular compartments (nucleus, granules, cytoplasm/cytoskeleton, enzymes, and plasma membrane) were identified to build a custom “NETosis” pathway. Profile plot from the Gene Set Enrichment Analysis (GSEA) showing highly significant enrichment of most proteins in the custom NETosis pathway in the 4 replicates (TAAD1-3, TBAD 4) compared with negative control subjects (CTRL1-2). The false discovery rate–adjusted P value for this analysis, according to GSEA output, was < 0.01. **(B)** The expression of granules and nuclear NET components was significantly higher, and non-granular enzymes and membrane components were mildly enriched in aortic tissue from AAD patients. **(C)** Neutrophil elastase was significantly higher in aortic tissue from the AAD group than in aortic tissue from the control group. **(D,E)** The elastase level in patients with in-hospital death or without 1-year survival was significantly higher than that in patients without in-hospital death or with 1-year survival. ^*^P < 0.05, ^**^P < 0.01.

The expression levels of granule and nuclear NET components were significantly higher, and non-granular enzymes and membrane components were mildly enriched in aortic tissue from AAD patients compared with normal controls (Figures 5A,B). The expression of neutrophil elastase, the prototypical NET marker, was significantly higher in aortic tissue from the AAD group than in aortic tissue from the control group (Figure 5C). Additionally, the elastase level in patients with in-hospital death or without 1-year survival was significantly higher than that in patients without in-hospital death or with 1-year survival (Figures 5D,E).

**Figure 6:**
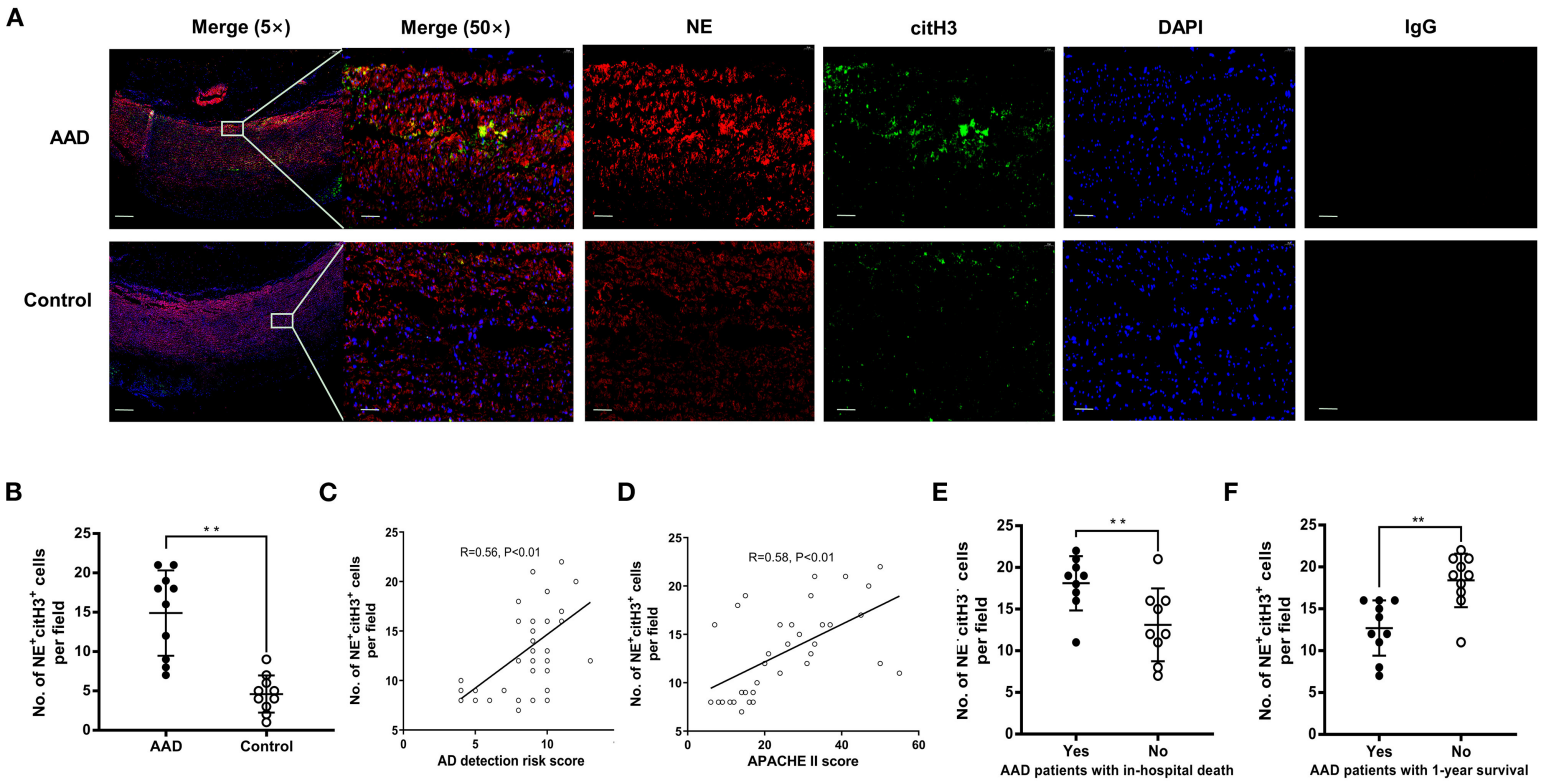
The association between excess NETs in aortic tissue and disease severity or prognosis of patients with AAD. **(A)** Local NETosis was detected in aortic samples from AAD patients, as colocalization of NE with citH3 was observed by confocal microscopy. NE was stained green (Alexa Fluor 488) and citH3 was stained red (Alexa Fluor 647). DAPI staining was used to mark nucleus (blue fluorescence). **(B)** Compared with normal controls, the number of NETs formed per field in the tissue samples from AAD patients was significantly increased. **(C,D)** The number of NETs formed per field was positively correlated with the detection risk score of AD and the APACHE II score. **(E)** The number of NETs formed per field in patients with in-hospital death was significantly higher than that in patients without in-hospital death. **(F)** The number of NETs formed per field in patients with 1-year survival was significantly lower than that in patients without 1-year survival. ^**^P < 0.01.

Local NETosis was detected in the aortic samples from AAD patients, as colocalization of NE with citH3 was observed by confocal microscopy (Figure 6A). Compared with healthy donor aortic tissue, the numbers of NETs formed per field in the tissue samples from AAD patients were significantly increased (Figure 6B). Moreover, the number of NETs formed per field was positively correlated with the detection risk score of AD and the APACHE II score (Figures 6C,D). The number of NETs formed per field in patients with in-hospital death or death within 1-year was significantly higher than that in patients without in-hospital death or death within 1-year (Figures 6E,F). These results indicated that excess NET components in aortic tissue samples are associated with the disease severity and prognosis of AAD.

## Discussion

To the best of our knowledge, this is the first time that the diagnostic and prognostic value of NETs has been evaluated in patients with AAD. In this study, the major findings were that: (a) circulating NET markers had reliable diagnostic value of AAD, with superior diagnostic performance to discriminate patients with chest pain from other reasons in the emergency department compared with D-dimer; (b) circulating NET markers were independently associated with the disease severity, in-hospital death and 1-year survival of patients with AAD; and (c) excessive NET components in the aortic tissue were associated with the disease severity and prognosis of AAD patients.

It is well-known that neutrophils are the first line of defense against infections by pathogens. Neutrophils are also pivotal as primary effector cells at sites of inflammation, but through regulation of their survival by means of regulated cell death, these cells are also involved in the resolution of inflammation (19). Significant neutrophilic infiltration was found not only in early but also in later stages of organizing dissections and not only in the clot but also in adventitial fat (10, 20). This could reflect an upregulation of neutrophil survival to maintain the intense tissue remodeling required for the repair of the arterial wall. In both surgical and autopsy cases of AAD, immunohistochemical staining for citH3 revealed a massive presence of NETs in the clot and in the adventitia in the subacute stage and less abundantly in early organizing stage dissections (10). It is worth noting that aortic tissue samples were collected during surgery in this study. However, in surgical cases, awareness of the pattern of margination, transmigration and extravasation around microvessels that may occur due to robust handling during surgery should be considered to avoid overinterpration of the more diffuse patterns of neutrophil infiltration and NET release related to dissection.

Our results elucidated that the three markers of NETs are significantly elevated in patients with AAD compared with the corresponding levels in subjects from the control group. Recent reports have documented the association of NETs with the severity of stroke evaluated by clinical indexes (21, 22). Our study showed for the first time that the three markers of NETs were in line with the disease severity score of AAD at onset and discharge. However, the APAHE II scoring system is predictive of disease severity only when specific baseline and wound characteristics are accounted for. A plasma marker that can be easily and quickly tested to stratify patients with different risks of mortality is of great prognostic significance.

In this study, a time course study of NET markers was performed in AAD patients according to the admission time from symptom onset. The levels of all three NET markers peaked within 12–24 h after symptom onset. However, the concentrations of NET markers were measured using leftover samples of the patients from initial presentation. However, strictly speaking, the time after the actual onset of disease might be different for each patient. It would be beneficial to study the changes in concentrations according to time course or disease progression intraindividually. In addition, the time window for NET concentrations returning to the baseline level after surgery also needs to be determined. This will provide a stable time horizon for the testing of NET markers to predict the outcome of treatment for patients with AAD.

The mechanism underlying the increased NET formation in AAD remains unknown. AAD is considered an active inflammatory process that occurs in response to endothelial damage through high shear stress. Neutrophils and NETs are emerging as important mediators of pathogenic inflammation in the aorta (23). When aortic dissection occurs, disruption of the aortic media immediately changes aorta hemodynamics, with intramural hemorrhage leading to propagation and tracking of blood within the media, which will overactivate the coagulation system. Currently, there is increasing awareness that NETs are linked to thrombosis since they may shift the hemostatic balance toward excessive coagulation (24). AAD exhibits high concentrations of tissue factor (TF) in serum, and the ability of neutrophils to expose functional TF on NETs is considered a link between inflammation and coagulation (25, 26).

In the last two decades, much progress has been made to make effective biochemical diagnoses of AAD, which is an unmet need with lifesaving value. Several promising biomarkers have emerged. Vinculin, lumican, MMP-12 and high levels of ischemia-modified albumin have been considered potential AAD-related serum markers that may assist in the diagnosis and prediction of the in-hospital mortality of patients with AAD (27–30). However, most of these biomarkers are still clinically unavailable. In real-world clinical practice, in patients with acute chest pain and elevated D-dimer, a diagnosis of AAD should be considered. D-dimer might be a useful complementary tool to the current diagnostic work-up of patients with suspected AAD (31, 32). D-dimer levels may be useful in risk-stratifying patients with potential AD to rule out AD if used within the first 24 h after symptom onset (33). Nevertheless, D-dimers are not always elevated in patients with AAD (34). The results of this study demonstrated that circulating NET markers showed significantly superior diagnostic performance compared with D-dimer to discriminate AAD patients with chest pain due to other reasons.

Despite acceptable reproducibility, as indicated by interassay cutoff values, the variability of the data makes clinically relevant cutoffs infeasible, and causality cannot be addressed. Although we used methods in line with current standards, the observed results highlight the need for improved methods when quantifying circulating NET markers (35). However, the inconsistent findings among the levels of cfDNA, citH3 and nucleosomes call into question their specificity in terms of reflecting NETosis. Previous studies have used various methods for evaluating the suitability of NETs as biomarkers in different clinical conditions, in which the main analytical targets were cfDNA, histones, and other components of NETs, such as neutrophil elastase or myeloperoxidase in plasma (36). cfDNA has repeatedly been described as a NET marker due to the objectivity of DNA quantification methods, yet its source is ambiguous, as non-neutrophil cells also release chromatin through cell death processes (35). The citrullination of histone 3 is a necessary step in the formation of NETs, as demonstrated by genetic and pharmacological approaches (37). Thus, citH3 appears to be the most specific marker of NETs, and it has been used to test for the presence of NETs in plasma. Importantly, citH3 has been reported to be independently associated with all-cause mortality during the 1-year follow-up in patients with acute ischemic stroke (21). In this study, citH3 was also identified as an independent risk factor for 1-year survival in patients with AAD.

Some limitations should be considered in the interpretation of our results. First, this is a single-center study with a relatively small number of subjects. Potential selection biases are not negligible. Future research on this topic should aim to include a larger study population. Secondly, a long-term follow-up period would be necessary to obtain statistically significant results in the prediction of disease prognoses. In this study, we reported major outcomes observed during the 1-year follow-up. Analysis of the changes in circulating levels of NETs during the follow-up period is also meaningful. Thirdly, our study was mostly descriptive, whereas the specific mechanism of NETs in circulation or aortic tissue promoting the occurrence and progression of AAD was not addressed in this study. Further research is needed to determine whether NET components could be used as potential therapeutic targets for AAD.

In conclusion, the present study demonstrates that circulating NET markers have significant diagnostic value for AAD with good diagnostic performance to discriminate patients with chest pain from other causes. NET markers, in both the serum and aortic tissue, are associated with disease severity and the prognosis of AAD patients at the 1-year follow-up. Our results suggest that NETs may constitute a useful diagnostic and prognostic marker in patients with AAD and open new avenues for future drug therapy for AAD.

## Data Availability Statement

The raw data supporting the conclusions of this article will be made available by the authors, without undue reservation. The data presented in the study are deposited in the (http://proteomecentral.proteomexchange.org) repository, accession number (PXD001603).

## Ethics Statement

The studies involving human participants were reviewed and approved by the Ethical Committee of Renji Hospital, School of Medicine, Shanghai Jiaotong University. The patients/participants provided their written informed consent to participate in this study.

## Author Contributions

Study conception and design and drafting of article were performed by SY and YX. Data collection was performed by JC and QN. Analysis and interpretation of data was performed by SY, YD, and XG. Critical revision was performed by GX and XX. All authors contributed to the article and approved the submitted version.

## Funding

This work was supported by Grants from the National Science Foundation of China (Grant No. 81700423 and No. 81873526), Clinical Research Innovation and Cultivation Fund of Renji Hospital (Grant No. PYIII-17-003), Shanghai Outstanding Young Doctor Training Program from Shanghai Municipal Commission of Health and Family Planning (to SY), Shanghai Jiaotong University Medical Engineering Cross Fund (Grant No. YG 2016QN57), scientific research project of Shanghai municipal commission of health and family planning (Grant No. 20164Y0058), Shanghai Jiao Tong University School of Medicine Nursing Research Key Project (Grant No. Jyhz1901), and Hangzhou science and technology development project (Grant No. 20201203B195).

## Conflict of Interest

The authors declare that the research was conducted in the absence of any commercial or financial relationships that could be construed as a potential conflict of interest.

## Publisher's Note

All claims expressed in this article are solely those of the authors and do not necessarily represent those of their affiliated organizations, or those of the publisher, the editors and the reviewers. Any product that may be evaluated in this article, or claim that may be made by its manufacturer, is not guaranteed or endorsed by the publisher.

## Abbreviations

AAD: acute aortic dissection
AMI: acute myocardial infarction
AD: aortic dissection
PE: pulmonary embolism
NETs: neutrophil extracellular traps
citH3: citrullination of histone 3
CTA: computed tomography angiography
MRA: magnetic resonance angiography
TAAD: type A aortic dissection
TABD: type B aortic dissection
APACHE II: the Acute Physiology, Age, Chronic Health Evaluation II
cfDNA: cell-free DNA
NE: neutrophil elastase
ROC: receiver operating characteristic curve
AUR: area under the ROC
NET: neutrophil extracellular trap
SBP: systolic blood pressure
TF: tissue factor.
