# Supplementary material for: Diagnostic and Prognostic Value of Neutrophil Extracellular Trap Levels in Patients With Acute Aortic Dissection
Source: Front Cardiovasc Med. 2022 Feb 15;8:683445. doi: 10.3389/fcvm.2021.683445 (PMC8885526; doi:10.3389/fcvm.2021.683445)
Supplement: Supplementary file 1 [file Table_1.DOCX]

**Supplemental Figure 1. Assessment of circulating NET markers in different subgroups.** (A) No significant difference in circulating NET markers was found between patients with TAAD and TBAD. (B) No significant difference in circulating NET markers was found among different subsets of the control group. (C) There was no correlation between age and circulating levels of NET markers. TAAD, type A aortic dissection. TABD, type B aortic dissection. * P < 0.05, ** P < 0.01.
