# Supplementary material for: Diagnostic and Prognostic Value of Neutrophil Extracellular Trap Levels in Patients With Acute Aortic Dissection
Source: Front Cardiovasc Med. 2022 Feb 15;8:683445. doi: 10.3389/fcvm.2021.683445 (PMC8885526; doi:10.3389/fcvm.2021.683445)
Supplement: Supplementary file 2 [file Table_2.DOCX]

**Supplemental Table 1. Baseline characteristics of healthy controls in this study**

| Characteristics | Healthy controls (n= 80) |
| --- | --- |
| Age, mean(range) | 49.1 (25-70) |
| Male sex, n (%) | 40 (50.0) |
| Biochemical and hematological data, mean ± SD | |
| Glucose, mg/dL | 92.3 ± 12.4 |
| Creatinine, μmol/L | 88.4 ± 13.6 |
| Uric acid, mg/dL | 4.9 ± 1.4 |
| Cholesterol, mg/dL | 118.3 ± 29.1 |
| Triglycerides, mg/dL | 122.1 ± 38.2 |
| AST, U/L | 18.2 ± 4.4 |
| ALT, U/L | 18.3 ± 5.3 |
| Bilirubin, mg/dL | 1.0 ± 0.4 |
| Hematocrit, % | 38.3 ± 5.4 |
| Platelets, 10^3^/μL | 215.4 ± 36.9 |
| Leukocytes, 10^3^/μL | 5.1 ± 2.3 |
| Lymphocytes, % | 28.2 ± 8.4 |
| Neutrophils, % | 67.3 ± 12.2 |
| Monocytes, % | 7.8 ± 3.3 |
| D-dimer, ng/ml | 212.4 ± 91.2 |

SD, standard deviation; AST, aspartate transaminase; ALT, alanine aminotransferase

**Supplemental Table 2. Diagnostic performance of patients with AAD versus others using markers of NETs compared with D-dimer**

| Variables | Threshold | AUR (95% CI) | Sensitivity, % | Specificity, % | Accuracy, % | PPV, %‡ | NPV, %‡ |
| --- | --- | --- | --- | --- | --- | --- | --- |
| citH3 | 0.25 A.U. | 0.87 (0.82, 0.93) | 73.96 | 100 | 92.75 | 100 | 90.88 |
| cfDNA | 403.5 ng/ml | 0.95 (0.93, 0.98) | 77.08 | 100 | 93.62 | 100 | 91.88 |
| nucleosomes | 0.51 A.U. | 0.92 (0.89, 0.96) | 81.25 | 100 | 94.78 | 100 | 93.26 |
| D-dimer | 2015 ng/ml | 0.64 (0.57, 0.71) | 57.29 | 84.74 | 55.61 | 59.14 | 83.73 |

citH3, citrullination of histone 3; cfDNA, cell-free DNA; AUR, area under the ROC curve; PPV, positive predictive value; NPV, negative predictive value

**Supplemental Table 3. Predictive value of 1-year survival using markers of NETs versus D-dimer in patients with AAD**

| Variables | Threshold | AUR (95% CI) | Sensitivity, % | Specificity, % | Accuracy, % | PPV, %‡ | NPV, %‡ |
| --- | --- | --- | --- | --- | --- | --- | --- |
| citH3 | 0.625 A.U. | 0.72 (0.61, 0.83) | 57.69 | 77.14 | 71.88 | 48.39 | 83.08 |
| cfDNA | 1052 ng/ml | 0.76 (0.66, 0.87) | 46.15 | 94.29 | 81.25 | 75.00 | 82.50 |
| nucleosomes | 2.155 A.U. | 0.73 (0.62, 0.85) | 61.54 | 82.86 | 77.08 | 57.14 | 85.29 |
| D-dimer | 915 ng/ml | 0.51 (0.38, 0.64) | 26.92 | 82.86 | 32.29 | 24.68 | 63.16 |

citH3, citrullination of histone 3; cfDNA, cell-free DNA; AUR, area under the ROC curve; PPV, positive predictive value; NPV, negative predictive value

**Supplemental Table 4. Univariate and multivariate risk factor analyses of 1-year survival of patients with AAD**

APACHE II, Acute Physiology and Chronic Health Evaluation II; citH3, citrullination of histone 3; cfDNA, cell-free DNA; SBP, systolic blood pressure

| Characteristics | Univariate Analysis | | Multivariate model | |
| --- | --- | --- | --- | --- |
|  | **HR(95% CI)** | **P value** | **HR(95% CI)** | **P value** |
| Gender | 1.589(0.691-3.657) | 0.276 |  |  |
| Elderly (>75y) | 1.949(0.847-4.485) | 0.117 | 0.608(0.223-1.872) | 0.372 |
| Hypertension | 0.743(0.323-1.708) | 0.484 |  |  |
| Diabetes | 0.713(0.323-1.571) | 0.401 |  |  |
| Stroke | 0.411(0.142-1.194) | 0.102 | 0.972(0.252-1.031) | 0.072 |
| Hyperlipidemia | 0.990(0.459-2.136) | 0.980 |  |  |
| Smoking | 1.024(0.470-2.229) | 0.953 |  |  |
| Marfan syndrome | 1.314(0.453-3.812) | 0.616 |  |  |
| Atrial fibrillation | 1.117(0.486-2.570) | 0.794 |  |  |
| Valvulopathy | 0.885(0.334-2.348) | 0.807 |  |  |
| APACHE II score | 1.022(0.725-1.441) | 0.901 |  |  |
| AD detection risk score | 1.031(0.730-1.455) | 0.863 |  |  |
| citH3 | 0.346(0.119-1.004) | 0.049 | 0.792(0.292-0.93) | 0.041 |
| cfDNA | 1.639(0.658-4.086) | 0.289 |  |  |
| nucleosomes | 1.101(0.479-2.533) | 0.820 |  |  |
| D-dimer | 0.830(0.333-2.068) | 0.689 |  |  |
| SBP | 4.96(0.314-0.786) | 0.003 | 0.496(0.314-0.786) | 0.003 |
