# Supplementary material for: Diagnostic and Prognostic Value of Neutrophil Extracellular Trap Levels in Patients With Acute Aortic Dissection
Source: Front Cardiovasc Med. 2022 Feb 15;8:683445. doi: 10.3389/fcvm.2021.683445 (PMC8885526; doi:10.3389/fcvm.2021.683445)
Supplement: Supplementary file 3 [file Table_3.DOCX]

**Proteomic profiles**

| Accession | Description | A5:105/104 | A5:107/106 | A5:106/104 | A5:106/105 | A5:107/104 | A5:107/105 | Average AD/CTRL ratio |
| --- | --- | --- | --- | --- | --- | --- | --- | --- |
| D6CHE9 | Neutrophil proteinase-3 OS=Homo sapiens GN=PRTN3 PE=2 SV=1 -[NPR3_Human] | 0.82 | 1.12 | 3.55 | 2.99 | 3.95 | 4.27 | 3.69 |
| B2MUD5 | Neutrophil elastase OS=Homo sapiens GN=ELA2 PE=4 SV=1 -[NE_Human] | 1.05 | 0.95 | 3.05 | 3.98 | 4.13 | 3.66 | 3.71 |
| Q16771 | Myeloperoxidase OS=Homo sapiens GN=MPO PE=4 SV=1 -[MPO_Human] | 0.98 | 1.02 | 3.79 | 3.21 | 4.06 | 3.55 | 3.65 |
| A0A161I202 | Lactoferrin OS=Homo sapiens GN=LTF PE=2 SV=1 -[LF_Human] | 0.95 | 1.05 | 3.05 | 4.12 | 2.86 | 3.26 | 3.32 |
| B2R4R0 | Histone H4 OS=Homo sapiens GN=HIST1H4J PE=2 SV=1 -[Histone H4_Human] | 1.11 | 0.89 | 2.25 | 3.97 | 4.26 | 2.32 | 3.2 |
| A4FTV9 | Histone H2A OS=Homo sapiens GN=HIST1H2AK PE=2 SV=1 -[Histone H2A_Human] | 1.13 | 0.87 | 2.3 | 3.83 | 3.82 | 3.13 | 3.27 |
| B2R4S9 | Histone H2B OS=Homo sapiens GN=HIST1H2BC PE=2 SV=1 -[Histone H2B_Human] | 1.05 | 0.95 | 3.62 | 4.01 | 2.23 | 3.01 | 3.22 |
| B2R4P9 | Histone H3 OS=Homo sapiens GN=H3F3B PE=2 SV=1 -[Histone H3_Human] | 0.86 | 1.14 | 2.3 | 3.89 | 3.56 | 3.9 | 3.41 |
| A0A0B4J1R6 | Transketolase OS=Homo sapiens GN=TKT PE=1 SV=1 -[TKT_Human] | 0.92 | 1.08 | 1.31 | 1.61 | 2.88 | 2 | 1.95 |
| A0A2U3TZU2 | GPI OS=Homo sapiens GN=GPI PE=1 SV=1 -[GPI_Human] | 0.75 | 1.25 | 2.94 | 0.65 | 2.54 | 0.31 | 1.61 |
| A0A3B3ITJ0 | Catalase OS=Homo sapiens GN=CAT PE=1 SV=1 -[CAT_Human] | 1.19 | 0.81 | 2.28 | 2.33 | 0.9 | 0.72 | 1.56 |
| A0A2R8Y879 | Alpha-enolase OS=Homo sapiens GN=ENO1 PE=1 SV=1 -[ENOA_Human] | 0.45 | 1.55 | 2.91 | 0.63 | 1.55 | 1.84 | 1.73 |
| E7EUT5 | GAPDH OS=Homo sapiens GN=GAPDH PE=1 SV=1 -[GAPDH_Human] | 1.07 | 0.93 | 1.96 | 1.95 | 2.22 | 1.83 | 1.99 |
| A0A087WSZ2 | Alpha-actinin OS=Homo sapiens GN=ACTN3 PE=1 SV=1 -[ACN_Human] | 1.24 | 0.76 | 1.19 | 1.18 | 1.17 | 0.99 | 1.13 |
| Q5TBN3 | Plastin-2 OS=Homo sapiens GN=LCP1 PE=1 SV=1 -[Plastin-2_Human] | 0.94 | 1.06 | 0.75 | 0.79 | 1.16 | 0.67 | 0.84 |
| Q60FE5 | Filamin-A OS=Homo sapiens GN=FLNA PE=1 SV=1 -[FLN-A_Human] | 0.67 | 1.33 | 0.95 | 0.65 | 0.66 | 0.69 | 0.74 |
| G5E9Q6 | Profilin-1 OS=Homo sapiens GN=PFN2 PE=1 SV=1 -[Profilin-1_Human] | 1.02 | 0.98 | 1.16 | 1.05 | 0.97 | 0.75 | 0.98 |
| B1AH99 | Myosin-9 OS=Homo sapiens GN=MYH9 PE=1 SV=1 -[MYH9_Human] | 0.76 | 1.24 | 1.09 | 0.98 | 1.07 | 1.1 | 1.06 |
| P68133 | Actin-1 OS=Homo sapiens GN=ACTA1 PE=N/A SV=N/A -[ACTA1_Human] | 1.14 | 0.86 | 0.83 | 0.75 | 1.08 | 0.82 | 0.87 |
| V9GZ54 | Moesin OS=Homo sapiens GN=MSN PE=1 SV=1 -[MYH_Human] | 1.21 | 0.79 | 0.94 | 0.7 | 0.8 | 0.93 | 0.84 |
| H6VRG1 | Cytokeratin-10 OS=Homo sapiens GN=KRT1 PE=1 SV=1 -[CK-10_Human] | 1.25 | 0.75 | 0.97 | 1.09 | 1.17 | 0.74 | 0.99 |
| B2ZDQ1 | NGAL OS=Homo sapiens GN=NGAL PE=2 SV=1 -[NGAL_Human] | 0.85 | 1.15 | 2.53 | 1.24 | 2.29 | 2.82 | 2.22 |
| Q5T3N1 | Annexin A1 OS=Homo sapiens GN=ANXA1 PE=1 SV=1 -[ANXA1_Human] | 0.76 | 1.24 | 0.99 | 0.86 | 1.11 | 2.57 | 1.38 |

The Table lists proteins that were identified in this study. Accession number and protein names are shown. AS 106/104, 106/105, 107/104, 107/105 are the internal iTRAQ technical replicates, while AS 105/104 and 107/106 are the internal swap technical controls.
