# Supplementary figures and images for: Diagnostic and Prognostic Value of Neutrophil Extracellular Trap Levels in Patients With Acute Aortic Dissection
Source: Front Cardiovasc Med. 2022 Feb 15;8:683445. doi: 10.3389/fcvm.2021.683445 (PMC8885526; doi:10.3389/fcvm.2021.683445)

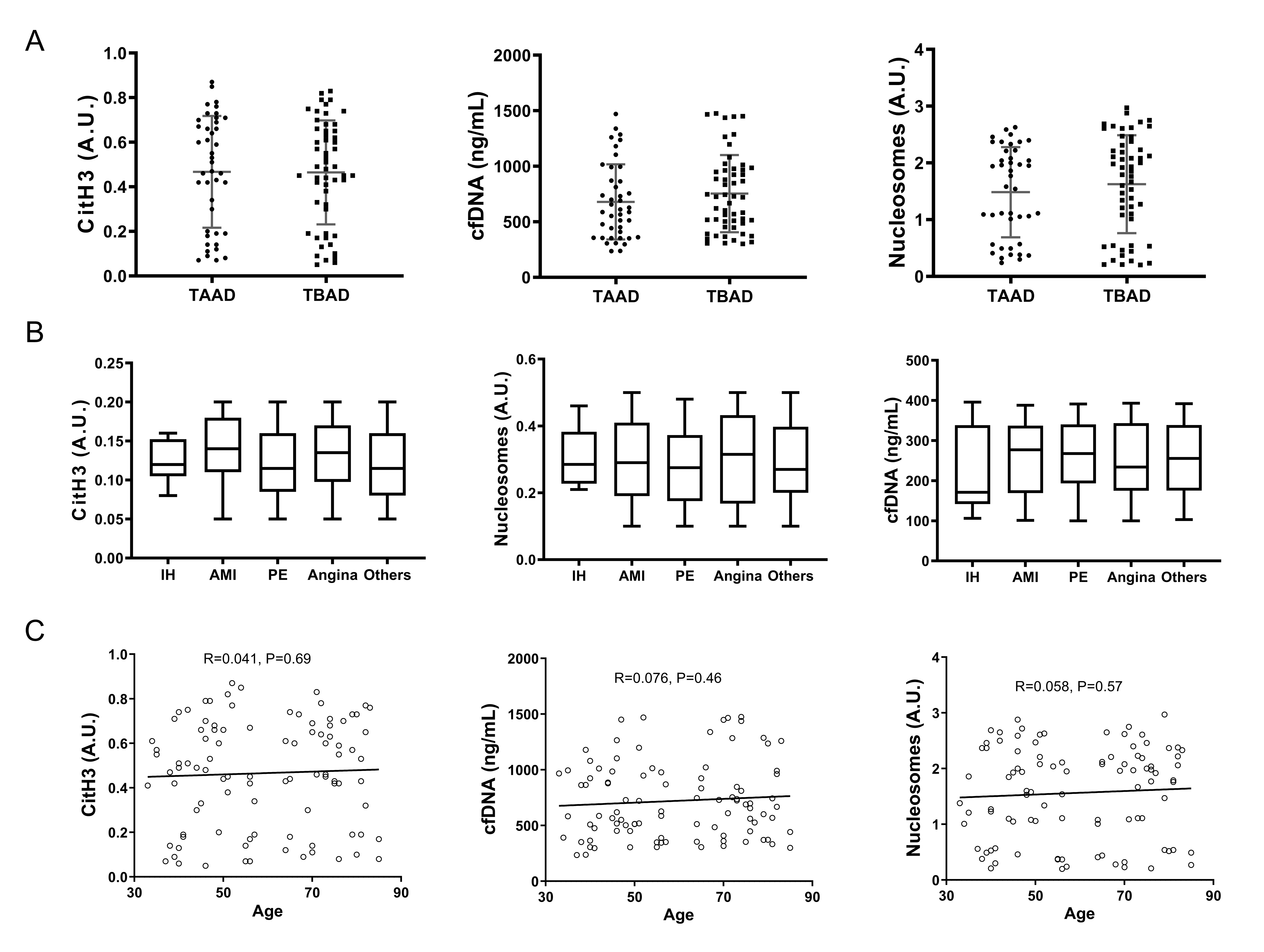

Supplement: Supplementary file 4 [file Image_1.TIF]
